# Effectiveness of multilevel interventions based on socio-ecological model to decrease sedentary time in children: a systematic review of controlled studies

**DOI:** 10.3389/fpubh.2023.1106206

**Published:** 2023-06-02

**Authors:** Marie Cholley-Gomez, Steven Laujac, Cyrille Delpierre, Marion Carayol

**Affiliations:** ^1^IAPS Laboratory “Impact of Physical Activity on Health”, University of Toulon, Toulon, France; ^2^ACTES Laboratory, University of Antilles, Pointe-à-Pitre, Guadeloupe; ^3^Centre Hospitalier Intercommunal Toulon-La Seyne sur Mer, Hôpital Sainte Musse, Toulon, France; ^4^EQUITY Team, CERPOP UMR 1295, Inserm-Université Toulouse III, Toulouse, France

**Keywords:** sedentary, intervention, prevention, socio-ecologic, multilevel

## Abstract

**Objectives:**

Preventive actions of sedentary behavior (SB) based on the socio-ecological model are needed among children and young adolescents. The aim of this systematic review is to ascertain the effectiveness of multilevel interventions (i.e., involving consideration of at least two interventional levels) in reducing sedentary time (ST) in children aged 5–12 years.

**Methods:**

Adhering to PRISMA guidelines, a systematic literature search was conducted in three databases (PsyInfo, PubMed and ERIC) until July 2021.

**Results:**

30 trials met the eligibility criteria and were included. They showed acceptable (< 8, *n* = 18) and high (≥ 8, *n* = 12) methodological quality. Among studies targeting 2 (*n* = 2), 3 (*n* = 19) and 4 levels (*n* = 9), 1 (50%), 9 (47%) and 7 (78%) were effective and reported significant reduction of ST, respectively.

**Conclusion:**

Interventions tend to be more effective when they involve 4 levels, using both agentic and structural strategies (targeting intrinsic determinants, in the organizational environment of the child). Findings underline the relevance of multilevel strategies to reduce ST in children, but also raise issues about operationalization of the socio-ecological perspective.

**Systematic review registration:**

PROSPERO, identifier: CRD42020209653.

## Introduction

### Sedentary behavior in young populations: an increasing public health concern

Sedentary lifestyle or sedentary behavior (SB) refers to “any waking behavior characterized by an energy expenditure ≤1.5 metabolic equivalents (METs)” (1), such as reading, watching TV, or working on a computer. Among SB, “screen-related” SB (2) are particularly worrying this last decade. Indeed, Sedentary Time (ST) has been associated with poorer health outcomes in children (3, 4).

However, a significant part of children and young adolescents does not reach active lifestyle recommendations (5): SB (i.e., ≥4 h 30 min of daily sitting time) was identified in 76.8% of European adolescents in 2017 with no differences between girls and boys (6), and over the world, 81% of adolescents aged 11–17 years were insufficiently physically active in 2016 (7). In France, national surveys showed that screen-time increases with age: more than 50% of school-aged children (6–10 years) spent at least 3 h/day [ESTEBAN 2014–2016 survey, see Balicco et al., (8)]; and between 2007 and 2015 [Inca2, 2007, and Inca3, 2015, see Dubuisson et al. (9)], screen time was increased by 20 min on average.

### The socio-ecological approach of sedentary behavior

There is a great demand in research for addressing public health issues by focusing on structural social determinants, particularly within the field of PA and sedentary lifestyle (10–14).

The socio-ecological model, based on the original work of McLeroy et al. (15) provides a useful comprehensive framework for this purpose. It marks a break with the cognitive behavioral-based approaches, by considering the social mechanisms of the production of health issues (16). The visual metaphor is a series of concentric circles representing different levels of influence on behavior. With a reciprocal determinism, each environmental level contains multiple types of environments (i.e., social, physical) and is in interaction with others.

Applied to the determinants of SB, this multifactorial approach states that these behaviors can be influenced by a multiplicity of levels, from the most proximal to the broader settings: intrapersonal [Psychological (e.g., self-esteem, attitudes toward SB) and physiological elements (e.g., capacities, health)], interpersonal [Social support of caregivers (parental rules, peers' behavior, encouragement from teachers…)], and organizational [Home; institution (care center, school): physical and social aspects (e.g., school wellness policy, garden equipment)] characteristics, and finally societal level including community Neighborhood, community environment (e.g., local associations) and public policies (Laws, national and local regulations (e.g., transport system, media, sports facilities in the city) (17).

The growing literature claiming for multilevel interventions assumed a larger effect on health outcomes, in comparison to single-level (intrapersonal) strategies but this argument suffers from limited empirical evidence (18–20).

### Interventions targeting sedentary lifestyle in school-aged populations

Preventive actions of SB are more and more needed among children and young adolescents [WHO guidelines, (5)]. Studies evaluating these actions in children have been increasing these recent years, and several systematic reviews and/or meta-analyses were published this last decade. Overall, these studies highlight the high heterogeneity of trials and the difficulty to establish strong evidence regarding interventions for the promotion of a less sedentary lifestyle. However, promising strategies are mentioned such as behavior change interventions (21, 22), electronic TV monitoring devices or TV turn-off (13, 23). Family and high parental involvement is a crucial interventional strategy (22, 24, 25), and, when focusing on school environment and policy, studies showed that adequate and accessible facilities for PA, and that educational materials, pedagogic practices and standing desks in classroom are significant opportunities in reducing ST (13, 23, 26, 27).

Multi-component and mixed interventions, incorporating both behavioral and environmental components (27, 28) were also mentioned as promising.

To this day, no study has systematically examined the effectiveness of multilevel, socio-ecological-based, interventions on ST-SB only, depending on the types and number of levels targeted by the strategies used. A few reviews have investigated socio-ecological or multilevel interventions specifically but none has focused on the reduction of ST/SB: Mehtälä et al. (29) investigated socio-ecological-based interventions aiming to increase the level of young (2–6 years) children's PA; the review of Kellou et al. (30) aimed to analyze the effectiveness of interventions preventing overweight in youngsters by promoting PA; in a recent review, Bernal et al. (20) compared the effectiveness of school-based multi-component vs. mono-component interventions carried out to promote children's PA.

Therefore, the aim of the present review is to systematically summarize evidence regarding the effectiveness of socio-ecological model-based multilevel intervention strategies to reduce ST in children and young adolescents. It aims to answer the following research questions: are interventions using multilevel/socio-ecological framework and targeting SB effective to reduce ST in children? Are these interventions more effective when they target more levels? In addition, as previously mentioned, to reduce ST, family-based interventions could be more effective if they use a strong parental involvement as a key strategy and not just as a supervisory role. This has led us to consider, in this review, not only the settings or the levels of the intervention, but also the involvement or not, and the degree of involvement of caregivers or social support surrounding the child (e.g., parents, teachers): are these multilevel interventions more effective when they involve a stakeholder/level representative (e.g., teacher, parent) at a strong degree?

## Methods

The present article reports a systematic review that has been conducted according to The Preferred Reporting Items for Systematic reviews and Meta-Analyses (PRISMA) guidelines. The review aims and methods were registered on PROSPERO (registration nr CRD42020209653).

### Systematic literature search and inclusion/exclusion criteria

Studies were included when they met the following PICOS criteria:

(i) P(Population): studies on healthy human subjects (i.e., no clinical population) that involve school-aged children (i.e., between 5 and 12 years-old) were included; studies with preschool < 5 years old and adolescents aged more than 12 were then excluded; studies involving only high-risk populations, defined as children or young adolescents being overweight with high risk of obesity, obese, or specific clinical populations (e.g., young with pathologies, e.g., cancer, or any disease) were excluded. Studies comparing normal weight children and obese children were included when results for the normal weight children were described separately.
(ii) I (Intervention): I (Intervention): intervention had to consider the reduction of ST, even if other health behaviors (e.g., nutrition habits, PA) could be mainly targeted; studies with interventions targeting at least two among the five levels of intervention according to the socio-ecological model of McLeroy et al. (15) (i.e., intra-, interpersonal, organizational, community-, society-based) were included;
(iii) C (Control), only studies with a control, or a comparison group (e.g., alternative intervention) were considered;
(iv) O (Outcome): studies had to report measures related to SB (e.g., TV viewing, computer-use, sitting-time); either objective (e.g., accelerometry) or reported (e.g., questionnaire) measures were considered.
(v) S (Study design): to be included, the study design had to test an intervention and to involve a comparative group. Randomized (or cluster randomized) studies were included but randomization was not mandatory. Studies performed in laboratory settings, studies without a control or comparative group, and cohort studies were excluded.

### Searching process

An electronic database search of PubMed (MEDLINE), PsycInfo and ERIC has been performed through the end of July 2021 (data published from 2000 to the present days, July 2021) languages restricted to English and French. We decided to start selection in 2000 as there has been growing consideration regarding ST and SB, and more particularly for a wide range of “screen-time related behaviors” (2) in these last two decades. Studies targeting only TV-viewing or computer-use seem to not accurately reflect a growing reality for children and young adolescents. Indeed, in young populations, most of the ST is made up of modern screen items that arose in the 2,000 decade (e.g., computer/laptop, smartphone, tablet (31). We used a combination of keywords related to sedentary lifestyle and screen-related behavior, public health interventions, preventive actions, and socio-ecological model, multilevel strategies or studies targeting several environments.

Finally, the research algorithm was the following: (sedentar^*^ OR screen^*^ OR multimedia) AND (intervention^*^ OR promot^*^ OR prevent^*^) AND (multi^*^ OR ecologic^*^ OR environment^*^ OR context^*^) NOT (disease^*^ OR patholog^*^). Limiters were the following: age ranging from 5 to 12 years; the study design: comparative, controlled, multicenter studies were included; the languages English and French; and the period of publication, starting from 2000 to July 2021.

First, the first author MCG selected eligible studies based on the title and/or the abstract and assessed the inclusion criteria to determine preliminary eligibility of studies. Following the PRISMA guidelines, at this first step of the selection on abstract, the author applied the PICOS method to check if the data fit the following inclusion criteria.

Second, MCG and MC separately read the full text, using the inclusion PICOS criteria to assess the final inclusion of articles. Any discrepancies were discussed until consensus was reached. MCG and SL extracted relevant data including methodology, participants, outcomes, and results. The following data were extracted: concerning the methodology, population details (country of intervention, number of and age mean or range of participants in control and intervention group), duration of intervention, use and type of theoretical framework, main setting (e.g., school, home) of intervention, study timelines. Each level targeted were identify; for the intrapersonal level, type of strategies (i.e., informational vs. behavioral) was detailed; in the interpersonal level, the type and degree of involvement (“+” if strong, meaning being active, “-” if rated weak, meaning passive) of caregivers (e.g., teachers, parents) were indicated. At the organizational level, type of setting was mentioned (e.g., school, home) with, for each of them, an indication of the kind of environment components (i.e., Physical, P, Social, S) targeted. Finally, results on SB-ST were briefly reported.

These elements are documented below in the summary **Table 2**, and described in results.

SL, PD, and MC checked the salient data and the methodological quality of trials included. Any discrepancies were resolved by discussion.

### Methodological quality

The methodological quality of each trial was examined using an 11-item scale derived from Cochrane collaboration's tools for assessing risk of bias in RCTs (81). This adapted scale, used by Gourlan et al. (82) in their review, assesses information of studies regarding (1) the eligibility criteria for participants; (2) the details of the intervention provided for each intervention level; (3) if the process of the intervention implementation was monitored; (4) the specific objective(s) of the study clearly mentioned; (5) the calculation technique used to determine the sample size was mentioned; (6) the method used to randomize participants [if randomization was used]; (7) the blinding to group assignment of assessors; (8) the participants flow; (9) the characteristics of the care providers performing the intervention; (10) the baseline data of participants are described for intervention and control groups; and (11) the number of participants included in each analysis is mentioned. All items were coded as “yes” (+), “no” (-) or “not applicable” (NA).

## Results

### Studies selection process

**Figure 1:**
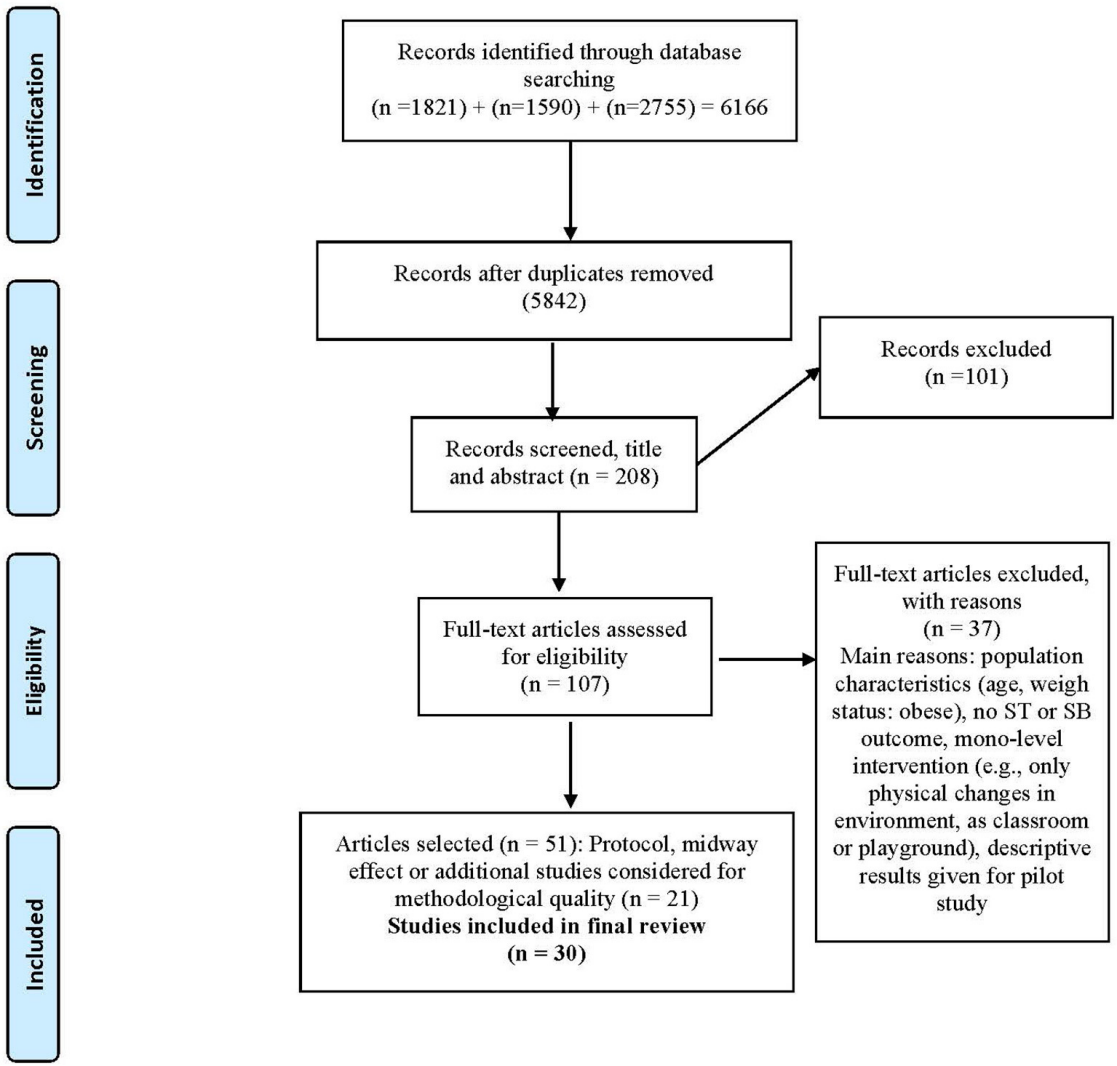
PRISMA flow diagram of the review (83).

The literature search yielded a total of 6,166 publications: 1,821 in Pubmed, 1,590 in ERIC and 2,755 in PsychInfo. The searching and selection process is summarized in the flow chart presented below, Figure 1. After removing duplicates (*n* = 5,842) and checking eligibility of the studies, a total of 30 relevant studies were finally included in this systematic review (reported by 51 publications, including e.g., protocol, midway, follow-up publications).

### Methodological quality

Briefly, based on the quality assessment form, most of the trials (*n* = 18 out of 30) demonstrated acceptable quality (i.e., rated under 8, on a total of 11 points), and 12 high quality (i.e., scoring ≥ 8). Concerning the criterion, the calculation technique used to determine the sample size of the trial was mentioned in less than half of the studies (*n* = 12 out of 30), and blinding to group assignment of assessors was mentioned in two trials only. Four studies were not randomized and among the others, 15 trials did not mention the method used to randomize participants. All studies clearly provided specific objectives, and most of them provided details of the intervention for each level (*n* = 28), participants' eligibility criteria (*n* = 21) and baseline characteristics (*n* = 29).

The following Table 1 summarizes the methodological quality assessment and reports the rate for each criterion and for each study selected.

**Table 1:** Methodological quality assessment of interventions selected for the review (detail by criterion and global quality score).

	**Eligibility criteria for participants**	**Details of the intervention provided for each level**	**Process of the intervention implementation was monitored**	**Specific objective(s) mentioned**	**Calculation technique used to determine the sample size mentioned**	**Method used to randomize participants**	**Blinding to group assignment of assessors**	**Participantsflow chart**	**Characteristics of the care providers performing the intervention**	**Baseline data of participants (Int/cont groups)**	**Number of participants included in each analysis**	**Qualityassessment score**
Breslin et al. (32)	+	+	-	+	-	NA	-	-	+	+	+	6
Carson et al. (33)	+	+	+	+	+	+	-	+	+	+	-	9
Duncan et al. (35)	-	+	+	+	-	-	-	-	-	+	+	5
Elder et al. (36)	+	+	-	+	-	-	-	-	+	+	+	6
Elder et al. (37)	+	+	+	+	-	-	-	+	+	+	+	8
Engelen et al. (38)	+	+	-	+	+	+	-	+	-	+	+	8
Escobar-Chaves et al. (40)	-	+	-	+	-	-	-	-	-	+	-	3
Folta et al. (41)	+	+	+	+	-	NA	-	+	+	+	-	7
French et al. (43)	+	-	+	+	-	-	-	-	+	+	+	6
Gentile et al. (45)	-	+	+	+	+	-	-	+	+	+	-	7
Harrison et al. (47)	+	+	+	+	-	-	-	-	+	+	-	6
Kattelmann et al. (48)	+	+	+	+	+	+	-	-	+	-	-	7
Kipping et al. (84)	-	+	+	+	-	+	-	+	+	+	+	8
Kipping et al. (50)	+	+	+	+	+	+	+	+	+	+	+	11
Kobel et al. (55)	+	+	+	+	+	-	-	+	+	+	+	9
Lynch et al. (56)	+	+	+	+	-	+	-	-	+	+	-	7
Madsen et al. (57)	+	+	+	+	+	-	-	-	+	+	+	8
Ni-Mhurchu et al. (59)	+	+	+	+	+	+	-	+	+	+	+	10
Nyberg et al. (60)	+	+	+	+	-	-	-	+	+	+	+	8
Pablos et al. (62)	+	+	-	+	-	+	-	-	+	+	+	7
Pearson et al. (63)	+	+	+	+	-	+	-	+	+	+	-	8
Salmon et al. (64)	+	+	+	+	+	+	-	+	+	+	-	9
Simon et al. (66)	+	+	+	+	+	-	-	-	+	+	+	8
Taylor et al. (70)	-	+	-	+	+	-	-	+	+	+	+	7
Todd et al. (71)	+	+	-	+	-	+	+	-	+	+	-	6
Van Kann et al. (72)	-	-	-	+	-	NA	-	+	+	+	-	4
Van Stralen et al. (73)	-	+	+	+	-	NA	-	-	-	+	-	4
Verloigne et al. (75)	-	+	+	+	-	-	-	-	+	+	+	6
Vik et al. (79)	-	+	+	+	+	-	-	-	+	+	+	7
Wright et al. (80)	+	+	-	+	-	-	-	+	+	+	+	7

### Characteristics of trials included

The salient data are summarized in Table 2 with a description of the participants' characteristics and main details of the intervention (duration, settings, theoretical framework, assessment methods, main results on ST-SB, strategies by level targeted, degree of caregiver's involvement).

**Table 2:** Main characteristics of trials and strategies by levels targeted.

**References study name, design [additional study]**	**Population details; Country**	**Duration**	**Theoretical framework**	**Main settings**	**Study timelines**	**Assessment of SB-ST**	**Results on SB-ST**	**Intrapersonal level (Informational, behavioral components)**	**Interpersonal level (social support) and Caregivers' involvement (strong +/weak -)**	**Organizational level (physical (P) and social (S) components)**	**Community level**
Breslin et al. (32) Sport for LIFE CT	*N =* 416 IG, n =209; Mage = 9.12 (0.37) CG, *N =* 207; Mage = 9.09 (0.35) low SES; Ireland	12 w	Social cognitive theory	School	Baseline and post	ST: accelerometers Screen behavior: Children survey	IG: ↓ SB (15 h-18 h and 18 h-bedtime)	Knowledge and behavior (class sessions and computer tailoring program; personal tailored feedback with specific suggestions to reduce screen-behavior)	Teachers (+) lead the implementation; principals and health nurses involved; Parents (+) (fact sheets informing and encouraging involvement in SB regulation + committees)	School and home (S)	
Carson et al. (33) Transform-Us! RCT [Salmon et al. (34)]	*N =* 293, 7 to 9y [SB] *N =* 74; [PA] *n =* 75; [SB+PA] *N =* 80; GC = 64; Australia	24m	Social cognitive and behavioral theory; ecological systems model	School and family	Baseline and midway (5–9 months)	ST: accelerometers	[SB + PA] group: ↓ ST in weekday	Knowledge and behavior key learning messages (class lessons) (e.g., social support, feedback); standing class lesson per day (30mn) and 2-min light active break	PE teachers (+): delivered content and active break, promoted PA at recess, made equipment available parents (-): newsletters supporting the key learning messages delivered	School and home (S and P environment: standing opportunities in classroom, PA equipment and asphalt line in playground)	
Duncan et al. (35) Healthy Homework RCT pilot study	*N =* 97; (57 IG, 40 CG) 9-11y; low SES; New Zealand	6w	Social cognitive/Behavior change theories	School and home	Baseline and post	Self-reported screen time: Children daily diary	No effect on SB	knowledge and behavior: homework booklet (5 PA and 5 nutrition topics) with reward, and in-class teaching resource; group presentations; Healthy Homework website	Teachers (+): active assistance, Parents (+) homework, tasks designed to encourage parental participation and knowledge formation	School and home (S)	
Elder et al. (36) Aventuras para Niños RCT	13 schools, 5-7y children low-income neighborhood; USA	1y	Social ecological model	School	Baseline and post	Direct observation: Researchers collected SOPLAY data	Supervised area; Area with equipment: ↑ number of boys engaged in SB (IG) Area organized activity: ↑ number of boys engaged in SB (CG	Behavior Trained ambassadors; “walking clubs”, “Super Aventuras” (activities options); incentives for participation (e.g., stickers, jump ropes, balls), training sessions	Parents (+): help for playground game marking Teachers (-): received feedback by the “promotoras” who led the implementation	School (S and P): line marking playground	
Elder et al. (37) MOVE/me Muevo RCT	541 families with children 5-8 y Public recreation centers in IG (*n =* 15), and control (*n =* 15); USA	2y	x	City recreation centers and Home	Baseline and post	ST: accelerometers	Non-significant differences on ST		Parents (+): household rules. 10-min telephone survey; 1$\frac{1}{2}$ hour group workshop with tip sheets (and by mail) at the recreation center, and a one-hour home visit. FU 10mn phone calls Recreation center directors (+): attendance of community members and enrollment of children in PA programs. Monthly meeting of recreational with intervention personal: action plan, monitor progress, and implement sustainable health policies	Home and recreation center (P and S aspects, e.g., healthy food and beverage offerings within the centers)	Health policies (recreational center) “community members”
Engelen et al. (38) It's child play RCT [Bundy et al. (39)]	226 children (5–7 y); Australia	13w	Intrinsic motivation	School (playground)	Baseline, post and post+2years	ST: accelerometers	IG: ↓ in sedentary activity during break times during breaktime: ↓SB (IG p=0,01) after 2 years: maintain of the gains		Teachers (-) and Parents (-): task and discussion: examine their own experiences and beliefs regarding the benefits and risks associated with active free play	School (S and P: loose materials for playground)	
Escobar-Chaves et al. (40) Fun Families RCT	202 families (101 int/GC) children 8.2 ± 0.8 y; USA	6m	Socio-cognitive theory; mapping intervention process	Home	Baseline and post	Parent's survey: media environment, media used by child and family screen habits	IG: less likely to report the TV being on when nobody was watching and to have a TV in the child's bedroom trend toward reducing actual media consumption but did not reach statistical significance	Knowledge and behavior discussions about puppet show (TV and media), creation of his hand puppet, brainstorming about alternative activities, make a healthy snack	Parents (+): 2-hour workshop (puppet show, interactive, and discussions) and 6 bimonthly newsletters. Behavioral objectives (e.g., no TV in the child's bedroom); Common work families and children: ≪ Fun family plan ≫ alternative activities	Home (S and P: no TV in bedroom)	
Folta et al. (41) CT Shape Up Sommerville; [Economos et al. (42)]	GI = 647; GC = 1074 6-8 y culturally diverse urban communities; USA	2y	Social Ecological Model	School, home and community	Baseline and post	Family survey form filled by parents/caregivers	IG ↓ screen time ↓ Overall screen time (-0,24h/day)	Knowledge and behavior taste tests with adult coordinators, who supervised the meal and modeled healthy eating. Walk to School Campaign	Parents (-): home environment was targeted through parent nutrition forums and newsletters	School (S and P: beverages provided for snack in the classroom, sold as a la carte snacks to meet nutritional guidelines)	Community environment: restaurants: alternative to sugar-sweetened beverage partnered with community members (+) (id, design and implement/evaluation)
French et al. (43) RCT, pilot study; NET-Works; [French et al. (44)]	IG: *N =* 25 GC: *n =* 15 (5-12 y); lower-income, minority and overweight children; USA	6m	Socio-ecological and behavior change	Home	Baseline and post	ST: accelerometers Parents survey reported child screen habit	IG: TV viewing alone (h/day) was lower than CG after 24 months (-16%) and 36 months (-12%); TV and computer use was lower than CG after 24 months	Behavior Work with parents and children to limit screen time on all devices	Parents (+): home visit and 5 monthly telephone calls, TV locking device with discussion and agreement; other small screens: work to limit child use and implement family home rules	Home (S and P: locking device on TV, non-caloric beverages given)	
Gentile et al. (45) RCT Switch program; [Eisenmann et al. (46)]	GC *n =* 674 GI: *n =* 685 Mage = 9.6 (0.9); USA	9m	Brofenbrenner's Ecological Model	School and community	Baseline, post and 6months FU	Screen time reported by both parents and children	Post-intervention: ↓parents reported screen time (persistency after 6 month)	Knowledge and behavior Identify healthy behavior, attitudes toward changeset (Do, view, Chew); achievement, short- and long-term goals; monthly: materials and resources to facilitate healthy target behaviors; behavioral tools to assist parents and children	Parents (+): identify health behaviors, resources and materials for behavioral change Teachers (+): materials and ways to integrate key concepts into their existing curricula not required to participate	Home, community and school (S)	public education intervention leadership group: leaders and project grantors from education, health care, government, business and the faith communities
Harrison et al. (47) “Switch Off—Get Active” RCT	*N =* 312 10.2 ± 0.7 y school social disadvantage area; Ireland	16w	Social cognitive theory for behavior change	School	Baseline and post	Self-reported “1-day previous day physical activity recall” survey (PDPAR)	IG: no difference in self-reported ST individual school analysis: ↓ Screen time: for 4/5 IG and 2/4 CG		Teachers (+) (10-lesson, teacher-led intervention) Parents (+) encouraged in writing to support children in their attempts &check behavior	School and home (S)	
Kattelmann et al. (48) iCook 4-H RCT; [Franzen-Castle et al. (49)]	228 youth (9-10y) –adult pairs; low-income and/or rural populations USA	12w	Social Cognitive Theory 4-H model of empowering youth	Home	Baseline and 4, 12, and 24 months	ST: accelerometers	IG: ST increased	Knowledge and behavior I-Cook 4H: curriculum about cooking, eating, and playing together for healthful lifestyles; website to share and interact	Parents (+): family activities; monthly newsletter that included the monthly challenge winners; Booster events: interact with other families (group playing games)	Home (S)	Community-based participatory research: Steering committees (research team, extension/4-H staff, Expanded Food and Nutrition Education Program staff (EFNEP), community members, and graduate students)
Kipping et al. (50) Active for life RCT, pilot study	*N =* 679 (9–10y); UK	5m	Social Cognitive Theory	School	Baseline and post	Screen based activities self-reported by questionnaire	IG: less time on screen-viewing than CG (non-significant). These differences were imprecisely estimated	Knowledge and behavior Lessons on healthy eating, PA and reducing TV viewing; games and activities	Teachers (+): led lessons	School (S)	
Kipping et al. (50) AFLY5 - AFLY5 RCT [Lawlor et al. (51, 52); Dreyhaupt et al. (53); Anderson et al. (54)]	*N =* 2221; 8-9 y; IG *N =* 1064; CG *N =* 1157; UK	1y	Social Cognitive Theory	School	Baseline, post and 1-year FU	ST: accelerometers Screen viewing: self-reported by questionnaire	No effect on objective ST; After taking account of multiple testing in analyses: effect on self-reported time spent on screen viewing at the weekend (Saturday) in IG	Knowledge and behavior lessons on school-time (contents promoting PA, healthy nutrition, and strategies to achieve healthy behaviors) and games (same topics), family activities at home	Parents (+): newsletter and homework parent-child interactive homework activities (e.g., “freeze my TV”, alternative active activities) Teachers (+): 16 lesson plans and teaching materials	School and home (S)	
Kobel (55) “Join the Healthy Boat” RCT	*N =* 1736; IG: *N =* 954; CG: *N =* 782 (7.1 ± 0.6 years); Germany	1y	Social Cognitive Theory	School	Baseline and post	Screen media use (SMU): parental questionnaire	SMU: IG: for girls, children without a migration background and children whose parents have low education level: ↓ screen media use by day.	Knowledge and behavior lessons (curriculum) and teaching materials offering action alternatives for recreational activities without screen media, PA, and a healthy diet + website to interact	Teachers (+): led lessons	School (S)	
Lynch et al. (56) Let's Go! 5-2-1-0 RCT, pilot study	*N =* 51, IG = 29, CG = 22 Mage = 8; USA	4m	x	School	Baseline and post	Reported ST: “Healthy Habits Survey” completed by caregiver	No statistical difference for ST	Knowledge key daily messages; Topics: weight, fruits and vegetables, recreational screen-time, PA, nutrition, sugary drink	Parents (-) and teachers (-): packet of information, prepared by the study team and sent home by teachers curriculum administered by nursing student, public health nurse, or a patient education specialist	School and home (S)	
Madsen et al. (57) Energy Balance 4 Kids EB4K with Play RCT [Myers et al. (58)]	*N =* 879 GI = 583 GC = 296 4th and 5th grade low-income,; USA	2y	x	School	Baseline, midpoint, and endpoint	ST: accelerometers	no difference for ST; *post-hoc* analyses stratified by grade: 4th-grade IG: ↓ school-day ST	Knowledge and behavior 12-week nutrition and energy balance education/PA curriculum Playworks coach structured recess activities before and during school hours to encourage active participation and led a PA session with individual classes every other week	Parents (+) and teachers (+): trained to implement Playworks games and classroom management strategies in PE sessions team of school staff and parents to implement classroom wellness policies and make improvements in school food	S and P school environment: classroom wellness policies/school food packaging equipment for the district's central kitchen	Partnerships with national organization Play works; afterschool sports leagues
Ni-Mhurchu et al. (59) RCT, pilot study	*N =* 29 IG *N =* 15; 10.4 ± 0.9 y; CG *N =* 14; 10.4 ± 0.9 y; New-Zealand	6w	x	Home	Baseline and post	Frequency and duration of TV watching self-report by questionnaire	Time spent watching TV ↓ by 4.2 h/week in the IG but difference not statistically significant. Both groups reported decreases in total ST, between-group differences were not statistically significant		Parents (+) discussion: use of the Time Machine in the household, ideas to manage TV watching (e.g., rules as no TV during meal times, moving the TV)	Home S and P (time monitor)	
Nyberg et al. (60) Healthy School Start RCT; [Nyberg et al. (61)]	*N =* 243, 6y and parents low to medium SES; Sweden	6m	Social cognitive theory	School and home	Baseline, post-intervention and at 6-months FU	ST: accelerometry SB: parent reported (EPAQ questionnaire)	Subgroup analyses showed a significant gender-group interaction: ↑ST in boys from IG	Knowledge and behavior classroom activities: children's knowledge, attitudes and preferences and parents' role modeling for healthy behaviors Homework activities with parents	Parents (+): brochure sent home: Health information facts and advice (e.g., PA, screen-time) Motivational Interviewing: provide support + choose goal (target child's diet or PA), agenda tool Teachers (+) led classroom activities (with teacher's manual), involved in material/tools development	School and home (S)	
Pablos et al. (62) Healthy Habits Program (HHP) RCT	*N =* 158; CG; *N =* 76; IG; *N =* 82 10-12y; Spain	8m	x	School	Baseline and post	SB self-reported by questionnaire (Inventory of Healthy Habits)	SB: no significant changes (goal of less than 2 hours of total ST was not achieved)	Knowledge and behavior Healthy habits (diet, PA sleep and hygiene) + physical exercise session with games and worksheets	Parents (+) and teachers (+): talks for parents and teachers about healthy habits for school children; worksheet to complete at home, had to be signed each week by the parents	School and home (S)	
Pearson et al. (63) Kids FIRST RCT, pilot study	ST and Snacking *N =* 21; ST *N =* 25 Snacking *N =* 14; CG *N =* 15 9-11y UK	12w	Social ecological and Social Cognitive theories of behavior change.	School and home	Baseline and post	Screen Time self-reported by questionnaire (adaptation of ASAQ)	↓children's school day and weekend day TV/DVD viewing and computer game use in the ST + Sn (snacking) and ST, self-reported smartphone use ↑; study was not powered to detect statistical changes	Knowledge and behavior Key learning messages (knowledge about ST/Sn outcomes) delivered in online child classroom lessons; homework activities/challenges; learning message to be positive role models to family and friends	Parents (+): 1 online session and a package of resources (e.g. newsletters) strategies to participate in healthy ST and/or consumption of healthy snacks, Guide on how to implement behavior modification social support: learning message to be positive role mdels	School and home (S)	
Salmon et al. (64) Switch-2-Activity RCT; [Salmon et al. (65)]	*N =* 1048 9-12 y; Australia	20w	Social cognitive and behavioral choice theories	School and home	Baseline, post and 18-months FU	Screen-based behaviors self-reported by survey	Screen based behavior: sex as moderator IG: ↓ST on week end for boys (-20min) self-efficacy reducing TV viewing: sex interaction IG > CG average change score IG: positive effect on boys and girls	Knowledge and behavior Introduction to AP and health; patterns of TV viewing and self-monitoring; selective TV viewing and behavioral contracting; identifying alternative activities and games; walking (pedometer) and games and activities developed by the children	Teachers (+) Delivering material (many teachers reported modifying it in some way)	School and home (S)	
Simon et al. (66) ICAPS RCT; [Simon et al. (67–69)]	*N =* 954 CG: *n =* 479 IG, *n =* 475 11-12y; France	4y	Socio-ecological model	School, home and community	Baseline, post and 2 years FU	ST Self-reported (adaptation of the MAQ questionnaire)	6months (Simon, 2006): proportion of IG adolescents spending > 3 h/day in sedentary occupations ↓ post: IG: ↓of TV viewing time (-16 min/day) FU (2014): differences in ST maintained	Knowledge and behavior objective: changing attitudes through debate and access to attractive activities during breaks and after-school hours, encouraging social support emphasis on fun, meeting with others and absence of competitive aspects	Parents, teachers, educators (+) social support: fostered to promote PA and to increase sports participation of children	school and home (S and P components) providing environmental conditions (e.g., accessibility) that enable PA	Event-specific activities numerous partnerships (medical staffs, PA and club educators, families, territorial and community agencies in charge of recreational areas and transportation infrastructures)
Taylor et al. (70) Active Schools: Skelmersdale (AS:Sk) pilot RCT	*N =* 232, 9–10 y CG: *n =* 115, IG: *n =* 117 low income; England	8w	Socio-ecological model, TEO, behavior change models	School	Baseline and post	ST: accelerometer	IG ↓ 9mn school ST	Behavior active breaks, bounce at the bell (suggested jump routine), ‘Born To Move' videos, Daily Mile or 100 Mile Club (challenge), playground activity challenge cards	Teachers (+) PE teacher training Parents (+): newsletters, homework activity and letters	School and home (S and P components: playground installations)	
Todd et al. (71) Pilot RCT	IG: *N =* 11 (10.0 ± 0.8) CG: *N =* 10 (9.7 ± 1.2) boys (excessive screen-use); USA	20w	x	Home	Baseline, midpoint (10w), endpoint	Electronic media time self-recorded on logbooks	10 weeks: IG: ↓ of electronic media use (-47%/day) and achieved target (< 90min/day); CG also ↓ (-24%) but exceeded the IG and the target (+29 min) At 20 weeks, IG media use remained 8 min/day below the target, CG 5min/day	Knowledge and behavior participants were matched in pairs; seminar designed to enhance awareness of electronic media use and to set goals to minimize use: family-centered interactive session,	Parents (+) follow-up daily with the children for completing data; interactive family session (TV), 3 newsletter (TV), follow-up phone call with recommendations	School and home (S and P components: monitor device on TV and computer)	
Van Kann et al. (72) Active Living project CT	*N =* 791 8–11 years; deprived areas Netherlands	1y	Ecological systems theory	School	Baseline and 1y effect	ST: accelerometer	IG:−5,9% in SB (nonsignificant) -female gender: significant predictor for more SB (follow up) -children in 7th grade: more time in SB (follow up) intervention components: More and higher intensity PSI= ↓SB (after 12 months)	Knowledge presence of posters in school	Parents (-) communication in parental newsletters Teachers (+): Schools were supported in implementing physical and social schoolyard interventions to stimulate children's PA, e.g., teachers introducing schoolyard games	School S and P (equipment for playground, working budget)	Working groups, chaired by a municipal health service employee to identify environmental changes needed
van Stralen et al. (73) Jump-In CT; [De Meij et al. (74)]	*N =* 600; GI *n =* 259 GC *N =* 341 Mage = 9.8 ± 0.7 y; disadvantaged areas; Netherlands	2y	Precede-proceed model, ecological and socio-cognitive theories	School, home	Baseline and post (20months)	self-reported TV-viewing behavior and computer use	Non effective in reducing TV-viewing or computer time	Knowledge and behavior Pupil follow up system, yearly monitoring instruments of PA, BMI and motor skills, personal workbooks for children and their parents with assignments to perform in class and at home and an instruction book for the school staff	Teachers/School staff (+) and parents (+) Parental information services including information meetings, courses and sport activities for parents	School and home S and P environment (e.g., offer of structural and easily accessible school sport activities)	Sports club and local municipalities (short term sports courses, sports competitions and PA game), coordinators and trainers of these local sports activities
Verloigne et al. (75) ENERGY project RCT, pilot intervention; Verloigne et al. (76, 77), Van Lippevelde, et al. (78)	*N =* 372 Mage = 10.9 ± 0.7 y IG= *N =* 141; CG: *N =* 231; Belgium	6w	Social ecological perspective	School	Baseline and post	ST: accelerometers	No differences in ST between IG and CG	Knowledge and behavior lessons: awareness and evaluation of sitting time, influencing factors at home, possibilities for activity breaks and active transportation, and Family Fun Event, brainstorming, homework and activities	Parents (+): newsletters to involve the parents; personalized messages and homework tasks to be completed at home Family Fun Event Teachers (+): six weeks lasting intervention was conducted by the teachers	School and home (S)	
Vik et al. (79) UP4Fun RCT	*N =* 3147 Mage = 11.2 IG: *N =* 1569 CG *N =* 1578 5 European countries	6w	Planned Promotion for Population Health and socio-ecological models	School	Baseline and post (short-term)	Screen time and breaking up sitting time reported by questionnaire, total ST and breaking up sitting time by accelerometry	No significant intervention effects on ST, neither for self-reported or accelerometer-assessed ST	Knowledge and behavior lessons: awareness and evaluation of sitting time, influencing factors at home, possibilities for activity breaks and active transportation, and Family Fun Event, brainstorming, homework and activities	Parents (+): newsletters; personalized and homework tasks Family Fun Event Teachers (+): six weeks lasting intervention was conducted by the teachers	School and home (S)	
Wright (80) Kids N Fitness RCT	*N =* 251; 8–12y urban, low-income neighborhoods; USA	4m	Community-academic partnered participatory research (CPPR)	School, home and community	Baseline, 4 months, and 12 months post	TV viewing/computer game playing self-reported by questionnaire	TV viewing significantly decreased (to 4 months); effect was sustained at 12 months for males only	Knowledge and behavior: weekly 90-min sessions, PA/SB, nutrition education/behavior modification, and family involvement creative ways to exercise in a non-structured exercise program	Educational staff (+): staff professional development in health promotion and parents (+) family involvement sessions, newsletters and involvement as “active community stakeholders”	School (S) (School Wellness Policy involving dietary changes, staff professional development	Partnerships with local community clinics; nurse, trained community health workers and PE specialist; active community stakeholders (academicians, school administrators, teachers, parents)

Briefly, the 30 trials were published between 2000 and 2020, in 2006 for the earliest and in 2020 for the most recent with 23 (77%) studies in the last decade. Eleven interventions were conducted in the USA, 15 took place in Europe (e.g., Poland, Sweden, France, Belgium), and 4 in New Zealand, or Australia. Populations from 10 trials were made up of low-income groups from deprived areas; one study (71) solely targeted boys. Baseline sample sizes ranged from 29 children in a pilot study (59), to 3,147 in a trial (79) involving young populations from five European countries. The duration of the delivered interventions ranged from 4 months in the pilot study of Lynch (2016) (56) to 30 months for the trial led by Wright (2013) (80).

Social-cognitive theories (of behavior change/motivation) (85, 86) and socio-ecological models (12, 15) are the most frequently theoretical backgrounds mentioned. However, most of the studies (*N* = 18) do not refer to the socio-ecological perspective, or any ecological anchoring, and six studies do not mention any theoretical background.

Among the 30 included studies, the main setting of intervention is school in 24 trials. More precisely, three studies targeted only the home environment, four interventions only the school environment, two studies involved home and community (city recreation center; participatory research) and almost half of the trials (*N* = 14) school and home. For the remaining studies (*N* = 7), interventions were implemented or involved both school, home and community (partnership with community stakeholders, e.g., medical staffs, community health workers, local municipalities, PA club educators, territorial and community agencies in charge of transportation infrastructures).

Reported outcomes included ST or SB for all of the studies, and in 28 (93%) trials, PA outcomes (e.g., steps, sport participation, MVPA) was measured as well; only two trials did not targeted PA: screen-time was assessed in addition to dietary variables, and beverage consumption and BMI (43, 63). Regarding sedentary assessment, 16 (53%) trials used only subjective assessment of ST-SB: 11 studies reported only self-declared assessment; parental/caregiver questionnaire only, and combined with self-reported measures, were respectively used in 4, and one (45) study; one trial (36) used observational data recorded by researchers. Objective assessment was used in 8 trials, that solely used a monitored or device-based method (e.g., pedometer, accelerometer). Finally, a combination of self-declared and device-based, and parent's and device-based assessments were reported in 3, and 2 studies, respectively.

### Intervention components and strategies by level targeted

Table 2 summarizes the main characteristics of trials, and, for each targeted level, the type of strategy delivered. For the interpersonal level, we also considered the stakeholders/caregivers involved, and the strength of their participation.

Strategies used to deliver interventions can be described according to each socioecological model level.

Regarding the individual level (e.g., intrapersonal characteristics, such as attitudes, intrinsic motivation, skills), strategies were informational (e.g., passive: curriculum school program is designed to include health promotion and recommendation components about SB, energy-balance). Children sometimes received an educational program with key learning messages concerning various health determinants. Indeed, several interventions chose to include a multi-component strategy in delivering healthy messages: lessons and information could concern ST, PA, nutrition, or other health behaviors [e.g., (35, 48, 50)]. In this case, when the intervention aims to combine the messages on SB and physical activity with other health information, in an attempt to be more effective, it can be not easy to determine which component or part of the strategy was effective in reducing specifically ST, or an outcome isolated. Few studies (56, 72) mentioned informational determinants or knowledge in the intrapersonal level: e.g., delivering key learning messages (topics about weight, vegetables, recreational screen-time), presence of posters in the school. Cognitive components of strategies delivered could include goal setting to reduce electronic media, brainstorming, action-plan to achieve healthy behaviors or strategies to find alternative games and activities to replace SB. Mainly behavioral components were used in three interventions (36, 43, 70): e.g., active breaks bounce at the bell, playground activity challenge cards, training sessions, work with parents and children to limit screen-time. Most studies (*N* = 21) used a combination of informational and behavioral strategies (e.g., key learning messages during school lessons and a light active break; behavioral tools to modify behaviors and material and resources to identify healthy behaviors).

At the interpersonal level, one intervention component repeated in several designs of studies was the involvement of caregivers. Social support strategies were operationalized with the participation of parents, or other significant caregivers. The social circle, composed of people closed to the child, could be passively or actively involved: caregivers involved were mainly teachers and/or parents, but in some trials, school staff as principals, educators, health nurses, recreation center directors were also associated in the interpersonal level of intervention. Involvement was rated as “weak” (-) when passive: e.g., teachers who did not lead the lessons, but who were present during the intervention, who sent some information to the child's parents, who just received feedback from the research team who led the implementation. When their involvement was rated as strong (+), teachers could conduct the intervention, participate in material or tools development. Parents who took an active part in the intervention could attend workshops/meetings with their children, had activities or homework tasks to complete with children, or followed educational/motivational sessions with them. These study designs posit that having a supportive family environment can promote the targeted behavior change and be effective in reducing children's ST. Among studies (*N* = 27) targeting parents as social support (i.e., at the interpersonal level), 22 actively or strongly involved them, other studies targeted parents but with a more passive strategy (e.g., informational, as sending newsletters). In most studies (*N* = 20), implication of parents is linked with an involvement of school staff to target the entire social support of children (e.g., teachers, PE educators, nurses, educational staff in health promotion, recreational directors). This involvement is active in 17 trials.

At an environmental level (e.g., organizational, or institutional), almost half of the studies (*N* = 14) reported some physical components targeted: as for example, changes in the home or school physical environment (e.g., removing TV from the child's bedroom, install an electronic TV monitoring device, provide equipment and resources for physical activities, draw an asphalt line in the playground). Many studies were school-based [one was also recreational center based, (37)], some of them with a combination of school and home components strategies; few studies also included partnership with local municipalities, non-governmental partners, community stakeholders and external professionals (in the shape of collaborators in the field of nutrition, health staff, local community clinics, associations, municipal health employer, local sport clubs). Some actions were thus implemented outside of the initial institutional setting (e.g., steering committees with community members, afterschool sport leagues, sport competitions organized by sports clubs and local municipalities, event-specific activities in the community).

Few studies were community-based with a participatory research design (41, 45, 48, 80). In their intervention, Folta and others (2014) (41) targeted home and recreational centers, and the community environment by working with restaurants across the city to provide healthier options (e.g., offering more low-fat dairy products); the authors used a social ecological and systems approach, using community-based participatory research and involving community members in the development and implementation of the intervention. Kattelmann et al. (48) also used a similar design and formed steering committees composed of members of the research team, Expanded Food and Nutrition Education Program (EFNEP) staff, community members, and graduate students. Simon et al. (66) proposed, at the community level of their intervention, event-specific activities and established numerous partnerships (i.e., with medical staff, club educators, territorial and community agencies in charge of recreational areas and transportation infrastructures).

### Effectiveness of interventions

Two studies targeted 2 levels (interpersonal and organizational), and all (100%) had high methodological quality (i.e., score equal to or higher than 8). Then, 19 studies targeting 3 levels (mainly intrapersonal, interpersonal and organizational), of which 14 (74%) had high methodological quality. Last, 9 trials used four-level strategies (i.e., intra-, interpersonal, organizational and community-based), of which 2 (22%) showed high quality, and 7 (78%) a lower quality score. No study achieved to target the macro-environment or public policies level (e.g., social and cultural norms via media, urban planning, transport system).

**Figure 2:**
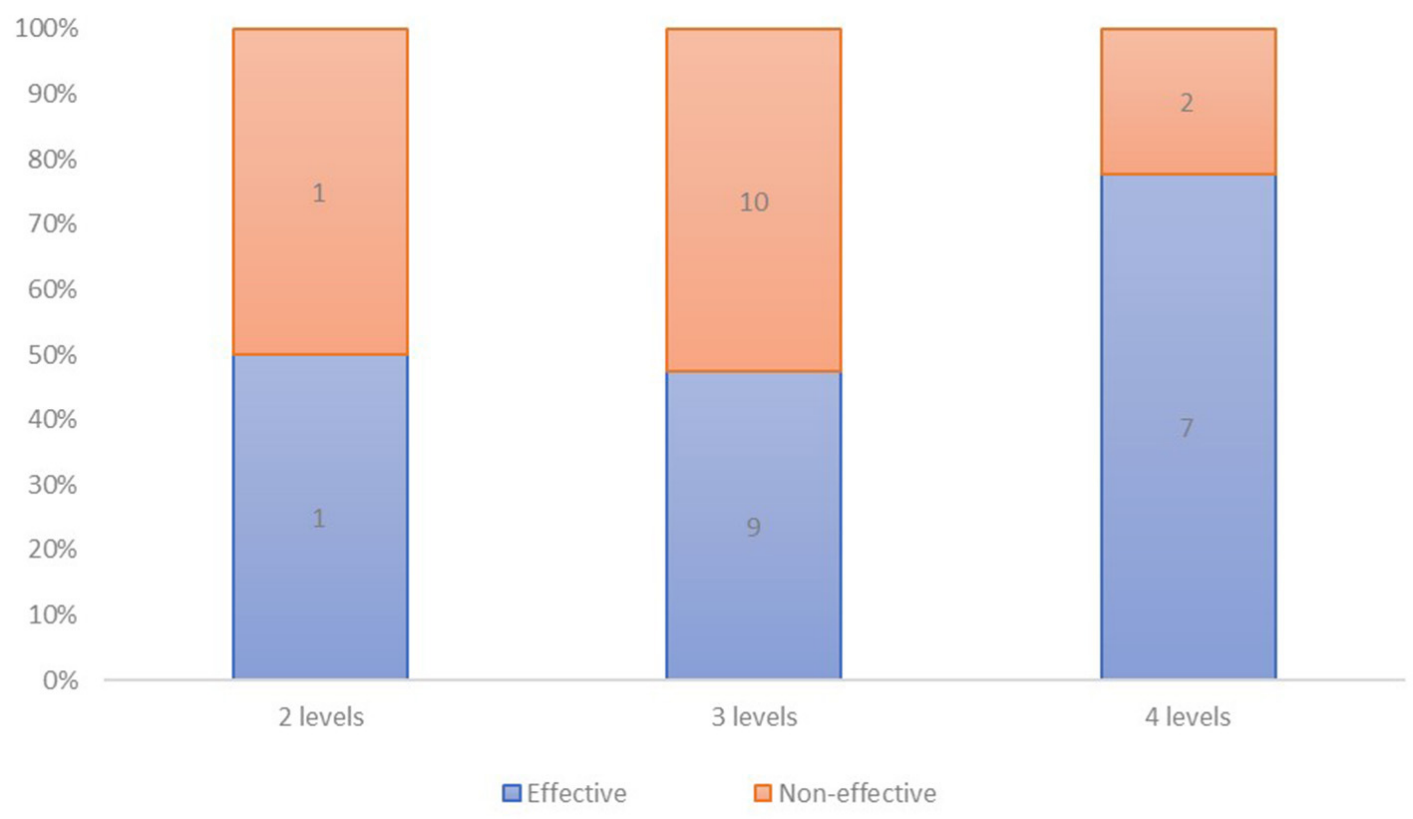
Number of interventions respectively reporting effectiveness and non-effectiveness on sedentary outcome, according to the number of levels targeted.

Effectiveness on sedentary outcomes was analyzed according to the number of levels targeted by the intervention, based on the socio-ecological model level stratification (see Figure 2). A trial was considered as effective when the study reported a significant impact of intervention on a sedentary measure at post- vs. pre-intervention; if several sedentary measures were reported and at least one showed a significant decrease of ST, the study was classified as effective.

As shown in the Figure 2, 1 (50%) of studies targeting 2 levels, 9 (47%) that targeted 3 levels and 7 (78%) trials that targeted 4 levels of intervention, were effective in reducing ST-SB.

Table 3 summarizes, for each trial, the effectiveness according to the number and type of levels targeted and to the methodological quality score.

**Table 3:** Effectiveness on ST-SB of trials according to methodological quality score, number and type of interventional levels targeted (as defined by the socio-ecological model).

**Number of levels targeted**	**Type of Levels targeted**	**Methodological quality score**			
		≥**8**		<**8**	
		**Effectiveness on ST-SB**	**No effectiveness**	**Effectiveness on ST-SB**	**No effectiveness**
2	Interpersonal and organizational	*N =* 1 Engelen et al. (38)	*N =* 1 Ni-Mhurchu et al. (59)		
3	Intrapersonal, interpersonal and organizational	*N =* 6 Carson et al. (33); Kipping et al. (50, 84); Subg: Kobel et al. (55)^a^; Nyberg et al. (60)^b^; Salmon et al. (64)^b^	*N =* 7 Escobar-Chaves et al. (40); Elder et al. (36); Harrison et al. (47); Lynch et al. (56); Pablos et al. (62); Verloigne et al. (75); Vik et al. (75)	*N =* 3 French et al. (43); Taylor et al. (70); Todd et al. (71)	*N =* 2 Duncan et al. (35); Pearson et al. (63)
3	Interpersonal, organizational and community		*N =* 1 Elder et al. (37)		
4	Intrapersonal, Interpersonal, organizational and community	*N =* 2 Simon et al. (66); subg: Madsen et al. (57)^c^		*N =* 5 Breslin et al. (32); Elder et al. (36); Folta et al. (41); Gentile et al. (45); Van Kann et al. (72); on subg: Wright et al. (80)^b^	*N =* 2 Kattelmann et al. (48); Van Stralen et al. (73)

^a^Gender and background migration; ^b^gender; ^c^grade.

As presented in Table 2, among the trials that used a 2-levels based intervention, all (*N* = 2, 100%) having high methodological quality, 1 (50%) was effective in reducing ST-SB. Among the high (*N* = 14) and the acceptable (*N* = 5) quality studies of the 3-levels trials, 6 (43%) and 3 (60%) were effective, respectively. Lastly, among the 2 high-quality and the 7 lower quality score interventions that used a four-level strategy, 2 (100%), and 5 (71%) were effective, respectively.

Regarding high quality studies, 9 (50%) reported effectiveness on ST-SB, and among those, 1 included 2-level, 6 involved 3-level, and 2 presented 4-level based interventions. Regarding lower quality studies, 8 (67%) reported effectiveness on ST-SB, and among those, none included 2-level, 3 involved 3-level, and 5 presented 4-level based interventions.

The second aim of our review was to consider the involvement, and the degree of involvement of caregivers or social support close to the child (involvement is reported in Table 2). Involvement was considered as strong when the stakeholder had an active role (e.g., a teacher-lead intervention during school class), and weak when only passive (e.g., parent receiving a newsletter with information about ST). Among the 22 studies targeting parents as social support with a strong or active involvement, 10 (45%) were effective in reducing ST-SB; among the 5 studies that targeted parents with a more passive strategy, 4 (80%) have reported effectiveness; in 3 of these trials, teachers, community members and recreational centers directors were also, and strongly, involved. Above the 30 included interventions, implication of parents is often associated with an involvement of educational stakeholders (e.g., teachers, educators in health promotion, educational staff, recreational directors, nurses). Among the 17 effective trials that considered social support as an intervention strategy, 2 (12%) involved parents only; 3 (18%) involved educational stakeholders only and 11 (70%) involved both parents and caregivers from the educational environment, one involving parents and community stakeholders.

Finally, among the 21 studies involving educational stakeholders (e.g., teachers, educators in health promotion, educational staff, recreational directors, nurses) with a strong or active involvement, 13 (62%) were effective in reducing ST-SB; among the 5 studies that included weaker involvement of educational stakeholders, 1 (20%) has reported effectiveness.

## Discussion

An important part of the scientific literature states that health behaviors linked with SB are influenced by intrapersonal factors, but also interpersonal and environmental determinants (11, 16, 17). Consequently, socio-ecological models and structural perspectives are drawing the attention of researchers (10, 13, 87). On a practical level, the combination of agentic and structural approach is operationalized by multi-level interventions that target multiple determinants, and use strategies at several levels of influence.

The purpose of this systematic review of the literature was to study and critically summarize controlled multilevel trials (i.e., targeting at least two levels of intervention) aiming to reduce SB (e.g., specific SB as TV-viewing, general ST), as primary or secondary outcome (e.g., it could first targeted PA) in young populations (children, from 5 to 12 years-old), and evaluate their effectiveness in relation to the number and the type of levels targeted (i.e., intrapersonal, interpersonal, organizational, and community), the methodological quality and the strategies (e.g., informational, behavioral, involving family and teachers) used in each level. To our knowledge, this is the first review that targets multilevel interventions specifically aiming for sedentary outcomes.

### Main findings

Final review included 30 controlled studies (most of them were published in the last decade) that involve 2, 19, and 9 interventions targeting 2, 3, and 4 different levels, respectively. Most of the included studies showed a high methodological quality score. However, in line with previous findings (22, 23, 30), the characteristics of included studies were heterogeneous, regarding the duration of the intervention (pilot studies had a shorter duration), population characteristics (e.g., size, age range, socioeconomic profile), settings (e.g., home, recreational center, school), assessment methods of ST-SB (i.e., subjective, objective, a combination of both measures), interventional strategies raised, leading to a difficulty to draw clear conclusions regarding the strategies and components that could preferentially reduce ST in children. Very few studies targeted 2 levels of intervention, also resulting in difficulty to make strong conclusions and to allow meaningful comparisons between 2-levels trials and other multilevel (i.e., 3- and 4-levels) studies. Most of the included multi-level interventions targeted 3 levels, mainly intrapersonal factors, interpersonal or social support level and organizational level (e.g., school, home). As young populations usually spend most of their time at school, this institution is a critical and major setting of interventions. Some interventions involved the community level, but none included study has achieved to target the society level (macro-environment).

Regarding effectiveness to reduce SB in children, effectiveness was reported in 1 (50%), 9 (47%) and 7 (78%) interventions targeting 2, 3, and 4 levels, respectively. Results suggest that interventions could be more often effective when the strategies used are deployed along 4 levels. However, only two studies used a 2-level strategy, providing insufficient evidence to rigorously conclude, and more than 70% of the 3-level studies have a high quality, whereas the majority of 4-level trials (78%) has an acceptable methodological quality.

This review secondly aimed to consider the involvement of caregivers in the effectiveness of trials. Again, the low number of trials and the heterogeneity of the interventions does not allow to drive specific and clear conclusions and, therefore, to answer this question. However, it seems that an involvement of caregivers (parents but also educational stakeholders, teachers in particular) could be a relevant strategy, targeting the entire social support of children. This design is based on the assumption that having a supportive family environment can promote the targeted behavior change and be effective in reducing children's ST. In this study, results show that among effective trials that considered social support as an intervention strategy, most of them (70%) involved both parents and educational caregiver or community members and among the studies strongly involving educational stakeholders, 62% were effective in reducing ST-SB.

Results ultimately suggest that the key ingredients of a successful intervention may involve a combination of several components: (i) at the intrapersonal level, both behavioral (e.g., setting screen-time goals) and informational strategies (e.g., often in the regular curriculum of the child), (ii) at the interpersonal level, a supportive and highly involved social circle by including teachers and parents, (iii) at the organizational level, targeting several child's environments (school and home) and (iv) at a community level including stakeholders (e.g., partnership with local sport clubs, local municipalities).

### Limitations and perspectives

Limitations of this work could be mentioned. First of all, a meta-analysis would have led to strongest conclusion. However, as previously raised, trials of very different nature met the inclusion criteria and were included and reviewed. Given the large heterogeneity in study design and intervention's characteristics, strong and relevant comparisons and analyses were difficult and it appeared that a meta-analysis was not relevant. This work also raised some methodological issues. The systemic approach proposed by the socio-ecological model is promising (10, 13), but also intimidating, integrating and conceptualizing different levels of the environment. Therefore, a challenge still remains in the *operationalization* of this model, such that *socio-ecological* is not a “buzzword” in public health (19), and in finding the methods for assessing the degree of integration of the socio-ecological model into research (87–89).

Future studies should analyze the impact of their intervention on ST-SB by specifying the type of SB (e.g., time spent reading, watching TV) and context (e.g., location or social situation). It is highly unlikely that a reduction in a specific SB will be directly replaced with PA; actually, it has a greater chance to be allocated to other SB (21, 22, 90).

When subjective assessments are used, different types of SB should be taken into account and lead to a comparison between different distinct behaviors (e.g., computer time vs. screen time) targeted by interventions (22). Domain-specific SB should be identified, with, as suggested by Owen et al. (13), a consideration of passive (e.g., TV viewing) vs. mentally active (e.g., reading, computer use) SB. Future trials should target other types of sedentary behavior, including non-screen-based measures, and consider the recent technological changes (3), given that this last decade, the use of small screens, as smartphones and tablets, is increasing in children.

In addition to the identification of domain-specific SB, there is a growing need to operationalize the distinction between passive (e.g., TV viewing) vs. mentally active (e.g., reading, computer use) SB (13). Moreover, the challenge of school-based interventions, even when they include home activities or home components in their strategies, is to reduce ST both at school and out of school. Another perspective thus concerns the need to interrupt ST during the whole day, as pointed out in the literature (30, 91, 92) and in the latest worldwide guidelines (5).

Targeting intrinsic levers in intervention strategies is important as only focusing on the environment of the child is not enough, considering that young people tend to select sedentary activities, even when physically active alternatives are available (22, 90, 93). On the other hand, targeting intrapersonal determinants to the detriment of the broader environment and of structural and political changes is an incomplete strategy. Future studies should use ecologic approach -e.g., targeting norms, physical components-, with a strong and active involvement of caregivers (social support) in the various environments (e.g., school staff, parents at home) of the child, in addition to curriculum or behavioral punctual strategies. Multilevel or socio-ecologic interventions should involve community level and the broader environment, as none included study has achieved to target the society level.

### Conclusion

A paradigmatic shift is occurring in the literature, and interventions targeting health behaviors are more and more multi-level or socio-ecological based. To our knowledge, no study had systematically reviewed and assessed the effectiveness of multilevel controlled trials targeting ST-SB in young (5–12 years) populations. Our findings show that more than half of the included interventions based on socioecological model (i.e., multilevel) have reported effectiveness of children SB. Indeed, among included studies, effectiveness on children SB was reported in 50%, 47%, and 78% interventions targeting 2, 3, and 4 levels, respectively. Therefore, results suggest that interventions could be more often effective when the strategies used are deployed along 4 levels. In addition, it seems that targeting four different levels i.e., intrapersonal, interpersonal, organizational and community, tend to led to more successful interventions to reduce SB.

This review highlights the need for additional randomized controlled trials evaluating multilevel interventions targeting ST-SB in young populations. More studies designing and implementing multilevel interventions are needed to “address the gap between theory and practice” (19) and remove operational and empirical hurdles. In addition, more reviews and meta-analyses are required to clearly assess their effectiveness and the key strategies underlying their effectiveness. Also, a theoretical and methodological reflection to quantify the degree of integration of the socioecological model in studies has to be continued to correctly evaluate the socio-ecological perspective.

## Data availability statement

The original contributions presented in the study are included in the article/supplementary material, further inquiries can be directed to the corresponding author.

## Author contributions

MC-G selected eligible studies and assessed the inclusion criteria to determine preliminary eligibility of studies based on the title and/or the abstract. Following the PRISMA guidelines, MC-G applied the PICOS method to check if the data fit the following inclusion criteria. MC-G and MC separately read the full text, using the inclusion PICOS criteria to assess the final inclusion of articles. Any discrepancies were discussed until consensus was reached. MC-G and SL extracted relevant data including methodology, participants, outcomes, and results. CD brought his expertise to this work and ensured the overall review of the manuscript. All authors contributed to the article and approved the submitted version.

## References

[1] Tremblay MS Aubert S Barnes JD Saunders TJ Carson V Latimer-Cheung AE . Sedentary behavior research network (SBRN) - terminology consensus project process and outcome. J Nutr Educ Behav. (2017) 14:75. 10.1186/s12966-017-0525-8 28599680 PMC5466781

[2] He M Piché L Beynon C Harris S. Screen-related sedentary behaviors: children's and parents' attitudes, motivations, and practices. J Nutr Educ Behav. (2010) 42:17–25. 10.1016/j.jneb.2008.11.011 19914872 PMC4898949

[3] Tremblay MS LeBlanc AG Kho ME Saunders TJ Larouche R Colley RC . Systematic review of sedentary behaviour and health indicators in school-aged children and youth. J Nutr Educ Behav. (2011) 8:98. 10.1186/1479-5868-8-98 21936895 PMC3186735

[4] Carson V Hunter S Kuzik N Gray CE Poitras VJ Chaput JP . Systematic review of sedentary behaviour and health indicators in school-aged children and youth: an update. Appl Physiol Nutr Metab. (2016) 41:S240–265. 10.1139/apnm-2015-0630 27306432

[5] Chaput JP Willumsen J Bull F Chou R Ekelund U Firth J . 2020 WHO guidelines on physical activity and sedentary behaviour for children and adolescents aged 5-17 years: summary of the evidence. Int J Behav Nutr Phys Act. (2020) 17:141. 10.1186/s12966-020-01037-z 33239009 PMC7691077

[6] López-Fernández J Lopez-Valenciano A Mayo X Liguori G Lamb M Copeland R . No changes in adolescent's sedentary behaviour across Europe between 2002 and (2017). BMC Public Health. (2021) 21:3. 10.1186/s12889-021-10860-3 33892700 PMC8067647

[7] Guthold R Stevens GA Riley LM Bull FC. Global trends in insufficient physical activity among adolescents: a pooled analysis of 298 population-based surveys with 1·6 million participants. Lancet Child Adolescent Health. (2020) 4:23–35. 10.1016/S2352-4642(19)30323-2 31761562 PMC6919336

[8] Balicco A Oleko A Szego E Boschat L Deschamps V Saoudi A . Protocole Esteban : une Étude transversale de santé sur l'environnement, la biosurveillance, l'activité physique et la nutrition (2014–2016). Toxicologie Analytique et Clinique. (2017) 29:517–37. 10.1016/j.toxac.2017.06.003

[9] Dubuisson C Dufour A Carrillo S Drouillet-Pinard P Havard S Volatier JL. The Third French Individual and National Food Consumption (INCA3) Survey 2014-2015: method, design and participation rate in the framework of a European harmonization process. Public Health Nutr. (2019) 22:584–600. 10.1017/S1368980018002896 30394264 PMC10260437

[10] Richard L Gauvin L Raine K. Ecological models revisited: their uses and evolution in health promotion over two decades. Annu Rev Public Health. (2011) 32:307–26. 10.1146/annurev-publhealth-031210-101141 21219155

[11] Simon C Kellou N Dugas J Platat C Copin N Schweitzer B . A socio-ecological approach promoting physical activity and limiting sedentary behavior in adolescence showed weight benefits maintained 2.5 years after intervention cessation. Int J Obesity. (2014) 38:936–43. 10.1038/ijo.2014.23 24509504 PMC4088336

[12] Sallis JF Owen N. Ecological models of health behavior. In: Health Behavior: Theory, Research, and Practice, 5th ed. Hoboken, NJ, US: Jossey-Bass/Wiley. (2015) p. 43–64.

[13] Owen N Healy GN Dempsey PC Salmon J Timperio A Clark BK . Sedentary behavior and public health: integrating the evidence and identifying potential solutions. Annu Rev Public Health. (2020) 41:265–87. 10.1146/annurev-publhealth-040119-094201 31913771

[14] Hu D Zhou S Crowley-McHattan ZJ Liu Z. Factors that influence participation in physical activity in school-aged children and adolescents: a systematic review from the social ecological model perspective. Int J Environ Res Public Health 18 mars. (2021) 18:3147. 10.3390/ijerph18063147 33803733 PMC8003258

[15] McLeroy KR Bibeau D Steckler A Glanz K. An ecological perspective on health promotion programs. Health Educ Q. (1988) 15:351–77. 10.1177/109019818801500401 3068205

[16] Wiltshire G Lee J Williams O. Understanding the reproduction of health inequalities: physical activity, social class and Bourdieu's habitus. Sport Educ Soc. (2019) 24:226–40. 10.1080/13573322.2017.1367657

[17] Sallis JF Owen N Fisher EB. Ecological models of health behavior. In: Health Behavior and Health Education: Theory Research and Practice 4th, ed. San Francisco, CA, US: Jossey-Bass. (2008) p. 465–85.

[18] Lieberman L Golden SD Earp JAL. Structural approaches to health promotion: what do we need to know about policy and environmental change? Health Educ Behav. (2013) 40:520–5. 10.1177/1090198113503342 24048612

[19] Schölmerich VLN Kawachi I. Translating the socio-ecological perspective into multilevel interventions: gaps between theory and practice. Health Educ Behav. (2016) 43:17–20. 10.1177/1090198115605309 26747715

[20] Bernal CMM Lhuisset L Fabre N Bois J. Promotion de l'activité physique à l'école primaire : évaluation de l'efficacité des interventions uni-leviers et multi-leviers. Mov Sport Sci/Sci Mot. (2020) 110:49–78. 10.1051/sm/2020022

[21] Biddle SJ O'Connell S Braithwaite RE. Sedentary behaviour interventions in young people: a meta-analysis. Br J Sports Med. (2011) 45:937–42. 10.1136/bjsports-2011-090205 21807671

[22] Biddle SJH Petrolini I Pearson N. Interventions designed to reduce sedentary behaviours in young people: a review of reviews. Br J Sports Med. (2014) 48:182–6. 10.1136/bjsports-2013-093078 24347578

[23] Altenburg TM. Kist-van Holthe J, Chinapaw MJM. Effectiveness of intervention strategies exclusively targeting reductions in children's sedentary time: a systematic review of the literature. Int J Behav Nutr Phys Act. (2016) 13:65. 10.1186/s12966-016-0387-5 27276873 PMC4899905

[24] Marsh S Foley LS Wilks DC Maddison R. Family-based interventions for reducing sedentary time in youth: a systematic review of randomized controlled trials. Obes Rev. (2014) 15:117–33. 10.1111/obr.12105 24102891

[25] dos Santos PC Barbosa Filho VC da Silva JA Bandeira A da S Minatto G da Silva KS. What works in sedentary behavior interventions for youth: a review of reviews. Adolescent Res Rev. (2019) 4:267–92. 10.1007/s40894-018-0105-424347578

[26] Morton KL Atkin AJ Corder K Suhrcke M van Sluijs EMF. The school environment and adolescent physical activity and sedentary behaviour: a mixed-studies systematic review. Obes Rev. (2016) 17:142–58. 10.1111/obr.12352 26680609 PMC4914929

[27] Hegarty LM Mair JL Kirby K Murtagh E Murphy MH. School-based interventions to reduce sedentary behaviour in children: a systematic review. AIMS Public Health. (2016) 3:520–41. 10.3934/publichealth.2016.3.520 29546180 PMC5689814

[28] Blackburn NE Wilson JJ McMullan II Caserotti P Giné-Garriga M Wirth K . The effectiveness and complexity of interventions targeting sedentary behaviour across the lifespan: a systematic review and meta-analysis. Int J Behav Nutr Phys Act déc. (2020) 17:53. 10.1186/s12966-020-00957-0 32334631 PMC7183680

[29] Mehtälä MAK Sääkslahti AK Inkinen ME Poskiparta MEH A. socio-ecological approach to physical activity interventions in childcare: a systematic review. Int J Behav Nutr Phys Act. (2014) 11:22. 10.1186/1479-5868-11-22 24559188 PMC3936868

[30] Kellou N Sandalinas F Copin N Simon C. Prevention of unhealthy weight in children by promoting physical activity using a socio-ecological approach: what can we learn from intervention studies? Diabetes Metab. (2014) 40:258–71. 10.1016/j.diabet.2014.01.002 24698814

[31] Arundell L Parker K Timperio A Salmon J Veitch J. Home-based screen time behaviors amongst youth and their parents: familial typologies and their modifiable correlates. BMC Public Health. (2020) 20:1492. 10.1186/s12889-020-09581-w 33004013 PMC7528232

[32] Breslin G Brennan D Rafferty R Gallagher AM Hanna D. The effect of a healthy lifestyle programme on 8–9 year olds from social disadvantage. Arch Dis Child. (2012) 97:618–24. 10.1136/archdischild-2011-301108 22685046

[33] Carson V Salmon J Arundell L Ridgers ND Cerin E Brown H . Examination of mid-intervention mediating effects on objectively assessed sedentary time among children in the Transform-Us! cluster-randomized controlled trial. Int J Behav Nutr Phys Act déc. (2013) 10:62. 10.1186/1479-5868-10-62 23688180 PMC3681598

[34] Salmon J Arundell L Hume C Brown H Hesketh K Dunstan DW . A cluster-randomized controlled trial to reduce sedentary behavior and promote physical activity and health of 8-9 year olds: the transform-Us! study. BMC Public Health. (2011) 11:759. 10.1186/1471-2458-11-759 21970511 PMC3213038

[35] Duncan S McPhee JC Schluter PJ Zinn C Smith R Schofield G. Efficacy of a compulsory homework programme for increasing physical activity and healthy eating in children: the healthy homework pilot study. Int J Behav Nutr Phys Act. (2011) 8:127. 10.1186/1479-5868-8-127 22085440 PMC3256102

[36] Elder JP McKenzie TL Arredondo EM Crespo NC Ayala GX. Effects of a multi-pronged intervention on children's activity levels at recess: the aventuras para niños study. Adv Nutr. (2011) 2:171S−176S. 10.3945/an.111.000380 22332049 PMC3065761

[37] Elder JP Crespo NC Corder K Ayala GX Slymen DJ Lopez NV . Childhood obesity prevention and control in city recreation centres and family homes: the MOVE/me Muevo project. Pediatric Obesity. (2014) 9:218–31. 10.1111/j.2047-6310.2013.00164.x 23754782 PMC3785546

[38] Engelen L Bundy AC Naughton G Simpson JM Bauman A Ragen J . Increasing physical activity in young primary school children—it's child's play: A cluster randomised controlled trial. Prev Med. (2013) 56:319–25. 10.1016/j.ypmed.2013.02.007 23462477

[39] Bundy AC Naughton G Tranter P Wyver S Baur L Schiller W . The Sydney playground project: popping the bubblewrap–unleashing the power of play: a cluster randomized controlled trial of a primary school playground-based intervention aiming to increase children's physical activity and social skills. BMC Public Health. (2011) 11:680. 10.1186/1471-2458-11-680 21884603 PMC3188492

[40] Escobar-Chaves SL Markham CM Addy RC Greisinger A Murray NG Brehm B. The fun families study: intervention to reduce children's TV viewing. Obesity. (2010) 18:S99–101. 10.1038/oby.2009.438 20107469

[41] Folta SC Kuder JF Goldberg JP Hyatt RR Must A Naumova EN . Changes in diet and physical activity resulting from the Shape Up Somerville community intervention. BMC Pediatr. (2013) 13:157. 10.1186/1471-2431-13-157 24093936 PMC3852296

[42] Economos CD Hyatt RR Goldberg JP Must A Naumova EN Collins JJ . A community intervention reduces BMI z-score in children: Shape Up Somerville first year results. Obesity (Silver Spring). (2007) 15:1325–36. 10.1038/oby.2007.155 17495210

[43] French SA Sherwood NE JaKa MM Haapala JL Ebbeling CB Ludwig DS. Physical changes in the home environment to reduce television viewing and sugar-sweetened beverage consumption among 5- to 12-year-old children: a randomized pilot study: Home decrease TV and SSBs. Pediatric Obes. (2016) 11:e12–5. 10.1111/ijpo.12067 26317968 PMC4833674

[44] French SA Sherwood NE Veblen-Mortenson S Crain AL JaKa MM Mitchell NR . Multicomponent obesity prevention intervention in low-income preschoolers: primary and subgroup analyses of the NET-works randomized clinical trial, 2012-2017. Am J Public Health. (2018) 108:1695–706. 10.2105/AJPH.2018.304696 30403521 PMC6236759

[45] Gentile DA Welk G Eisenmann JC Reimer RA Walsh DA Russell DW . Evaluation of a multiple ecological level child obesity prevention program: Switch^®^what you do, view, and chew. BMC Med. (2009) 7:49. 10.1186/1741-7015-7-49 19765270 PMC2758893

[46] Eisenmann JC Gentile DA Welk GJ Callahan R Strickland S Walsh M . SWITCH: rationale, design, and implementation of a community, school, and family-based intervention to modify behaviors related to childhood obesity. BMC Public Health. (2008) 8:223. 10.1186/1471-2458-8-223 18588706 PMC2474862

[47] Harrison M Burns CF McGuinness M Heslin J Murphy NM. Influence of a health education intervention on physical activity and screen time in primary school children: ‘Switch Off–Get Active'. J Sci Med Sport. (2006) 9:388–94. 10.1016/j.jsams.2006.06.012 16872900

[48] Kattelmann KK Meendering JR Hofer EJ Merfeld CM Olfert MD Hagedorn RL . The iCook 4-H study: report on physical activity and sedentary time in youth participating in a multicomponent program promoting family cooking, eating, and playing together. J Nutr Educ Behav. mars (2019) 51:S30–40. 10.1016/j.jneb.2018.09.002 30509553

[49] Franzen-Castle L Colby SE Kattelmann KK Olfert MD Mathews DR Yerxa K . Development of the iCook 4-H curriculum for youth and adults: cooking, eating, and playing together for childhood obesity prevention. J Nutr Educ Behav. (2019) 51:S60–8. 10.1016/j.jneb.2018.11.006 30851862

[50] Kipping RR Howe LD Jago R Campbell R Wells S Chittleborough CR . Effect of intervention aimed at increasing physical activity, reducing sedentary behaviour, and increasing fruit and vegetable consumption in children: active for Life Year 5 (AFLY5) school based cluster randomised controlled trial. BMJ. (2014) 348:g3256–g3256. 10.1136/bmj.g3256 24865166 PMC4035503

[51] Lawlor DA Jago R Noble SM Chittleborough CR Campbell R Mytton J . The active for life year 5 (AFLY5) school based cluster randomised controlled trial: study protocol for a randomized controlled trial. Trials. (2011) 12:181. 10.1186/1745-6215-12-181 21781344 PMC3158117

[52] Lawlor DA Peters TJ Howe LD Noble SM Kipping RR Jago R. The active for life year 5 (AFLY5) school-based cluster randomised controlled trial protocol: detailed statistical analysis plan. Trials. (2013) 14:234. 10.1186/1745-6215-14-234 23883177 PMC3733690

[53] Dreyhaupt J Koch B Wirt T Schreiber A Brandstetter S Kesztyüs D . Evaluation of a health promotion program in children: Study protocol and design of the cluster-randomized Baden-Württemberg primary school study [DRKS-ID: DRKS00000494]. BMC Public Health. (2012) 12:157. 10.1186/1471-2458-12-157 22394693 PMC3351371

[54] Anderson EL Howe LD Kipping RR Campbell R Jago R Noble SM . Long-term effects of the Active for Life Year 5 (AFLY5) school-based cluster-randomised controlled trial. BMJ Open. (2016) 6:e010957. 10.1136/bmjopen-2015-010957 27884840 PMC5168509

[55] Kobel S Wirt T Schreiber A Kesztyüs D Kettner S Erkelenz N . Intervention effects of a school-based health promotion programme on obesity related behavioural outcomes. J Obesity. (2014) 2014:1–8. 10.1155/2014/476230 25328688 PMC4190828

[56] Lynch BA Gentile N Maxson J Quigg S Swenson L Kaufman T. Elementary school–based obesity intervention using an educational curriculum. J Prim Care Community Health. (2016) 7:265–71. 10.1177/2150131916644888 27121724 PMC5932699

[57] Madsen K Linchey J Gerstein D Ross M Myers E Brown K . Energy balance 4 kids with play: results from a two-year cluster-randomized trial. Childhood Obes. (2015) 11:375–83. 10.1089/chi.2015.0002 26061799

[58] Myers EF Gerstein DE Foster J Ross M Brown K Kennedy E . Energy balance for kids with play: design and implementation of a multi-component school-based obesity prevention program. Child Obes. (2014) 10:251–9. 10.1089/chi.2013.0075 24783961

[59] Ni Mhurchu C Roberts V Maddison R Dorey E Jiang Y Jull A . Effect of electronic time monitors on children's television watching: Pilot trial of a home-based intervention. Prevent Med. (2009) 49:413–7. 10.1016/j.ypmed.2009.09.003 19744507

[60] Nyberg G Sundblom E Norman Å Bohman B Hagberg J Elinder LS. Effectiveness of a universal parental support programme to promote healthy dietary habits and physical activity and to prevent overweight and obesity in 6-year-old children: the healthy school start study, a cluster-randomised controlled trial. PLoS ONE. (2015) 10:e0116876. 10.1371/journal.pone.0116876 25680096 PMC4332680

[61] Nyberg G Ekblom O Marcus C. A 4-year cluster-randomised controlled intervention study on physical activity pattern and sedentary behaviour in children. Med Sci Sports. (2011) 43:24. 10.1249/01.MSS.0000402741.12322.4e27267965

[62] Pablos A Nebot V Vañó-Vicent V Ceca D Elvira L. Effectiveness of a school-based program focusing on diet and health habits taught through physical exercise. Appl Physiol Nutr Metab. (2018) 43:331–7. 10.1139/apnm-2017-0348 29136476

[63] Pearson N Biddle SJH Griffiths P Sherar LB McGeorge S Haycraft E. Reducing screen-time and unhealthy snacking in 9–11 year old children: the Kids FIRST pilot randomised controlled trial. BMC Public Health. (2020) 20:122. 10.1186/s12889-020-8232-9 31996192 PMC6988217

[64] Salmon J Jorna M Hume C Arundell L Chahine N Tienstra M . A translational research intervention to reduce screen behaviours and promote physical activity among children: Switch-2-Activity. Health Promotion Int. (2011) 26:311.21. 21177770 10.1093/heapro/daq078

[65] Salmon J Ball K Hume C Booth M Crawford D. Outcomes of a group-randomized trial to prevent excess weight gain, reduce screen behaviours and promote physical activity in 10-year-old children: switch-play. Int J Obes (Lond). (2008) 32:601–12. 10.1038/sj.ijo.0803805 18253162

[66] Simon C Schweitzer B Triby E Hausser F Copin N Kellou N . Promouvoir l'activité physique, lutter contre la sédentarité et prévenir le surpoids chez l'adolescent, c'est possible : les leçons d'ICAPS. Cahiers de Nutrition et de Diététique. (2011) 46:130–6. 10.1016/j.cnd.2011.03.003

[67] Simon C Schweitzer B Oujaa M Wagner A Arveiler D Triby E . Successful overweight prevention in adolescents by increasing physical activity: a 4-year randomized controlled intervention. Int J Obes (Lond). (2008) 32:1489–98. 10.1038/ijo.2008.99 18626482

[68] Simon C Wagner A DiVita C Rauscher E Klein-Platat C Arveiler D . Intervention centred on adolescents' physical activity and sedentary behaviour (ICAPS): concept and 6-month results. Int J Obes Relat Metab Disord. (2004) 28:S96–103. 10.1038/sj.ijo.0802812 15543228

[69] Simon C Wagner A Platat C Arveiler D Schweitzer B Schlienger JL . ICAPS: a multilevel program to improve physical activity in adolescents. Diabetes Metab. (2006) 32:41–9. 10.1016/S1262-3636(07)70245-8 16523185

[70] Taylor S Noonan R Knowles Z Owen M McGrane B Curry W . Evaluation of a pilot school-based physical activity clustered randomised controlled trial—active schools: Skelmersdale. IJERPH. (2018) 15:1011. 10.3390/ijerph15051011 29772839 PMC5982050

[71] Todd MK Reis-Bergan MJ Sidman CL Flohr JA Jameson-Walker K Spicer-Bartolau T . Effect of a family-based intervention on electronic media use and body composition among boys aged 8-−11 years: a pilot study. J Child Health Care. (2008) 12:344–58. 10.1177/1367493508097404 19052191

[72] Van Kann DHH de Vries SI Schipperijn J de Vries NK Jansen MWJ Kremers SPJ. A multicomponent schoolyard intervention targeting children's recess physical activity and sedentary behavior: effects after one year. J Phys Act Health. (2016) 1–28. 10.1123/jpah.2015-0702 27775465

[73] van Stralen MM de Meij J te Velde SJ van der Wal MF van Mechelen W Knol DL . Mediators of the effect of the JUMP-in intervention on physical activity and sedentary behavior in Dutch primary schoolchildren from disadvantaged neighborhoods. Int J Behav Nutr Phys Act. (2012) 9:131. 10.1186/1479-5868-9-131 23130806 PMC3541111

[74] De Meij JSB Chinapaw MJM Kremers SPJ Van der Wal MF Jurg ME Van Mechelen W. Promoting physical activity in children: The stepwise development of the primary school-based JUMP-in intervention applying the RE-AIM evaluation framework. Br J Sports Med. (2010) 44:879–87. 10.1136/bjsm.2008.053827 19019902

[75] Verloigne M Bere E Van Lippevelde W Maes L Lien N Vik FN . The effect of the UP4FUN pilot intervention on objectively measured sedentary time and physical activity in 10–12 year old children in Belgium: the ENERGY-project. BMC Public Health. (2012) 12:805. 10.1186/1471-2458-12-805 22989231 PMC3504538

[76] Verloigne M Berntsen S Ridgers ND Cardon G Chinapaw M Altenburg T . The UP4FUN intervention effect on breaking up sedentary time in 10- to 12-year-old belgian children: the ENERGY-project. Pediatr Exerc Sci. (2015) 27:234–42. 10.1123/pes.2014-0039 25389211

[77] Verloigne M Ridgers ND De Bourdeaudhuij I Cardon G. Effect and process evaluation of implementing standing desks in primary and secondary schools in Belgium: a cluster-randomised controlled trial. Int J Behav Nutr Phys Act. (2018) 15:94. 10.1186/s12966-018-0726-9 30261883 PMC6161341

[78] Van Lippevelde W Bere E Verloigne M van Stralen MM De Bourdeaudhuij I Lien N . The role of family-related factors in the effects of the UP4FUN school-based family-focused intervention targeting screen time in 10- to 12-year-old children: the ENERGY project. BMC Public Health. (2014) 14:857. 10.1186/1471-2458-14-857 25134740 PMC4150942

[79] Vik FN Lien N Berntsen S De Bourdeaudhuij I Grillenberger M Manios Y . Evaluation of the UP4FUN intervention: a cluster randomized trial to reduce and break up sitting time in european 10-12-year-old children. PLoS ONE. (2015) 10:e0122612. 10.1371/journal.pone.0122612 25826704 PMC4380348

[80] Wright K Giger JN Norris K Suro Z. Impact of a nurse-directed, coordinated school health program to enhance physical activity behaviors and reduce body mass index among minority children: a parallel-group, randomized control trial. Int J Nurs Studies. (2013) 50:727–37. 10.1016/j.ijnurstu.2012.09.004 23021318 PMC3654538

[81] Higgins JPT Altman DG Gotzsche PC Juni P Moher D Oxman AD . The Cochrane Collaboration's tool for assessing risk of bias in randomised trials. BMJ. (2011) 343:d5928–d5928. 10.1136/bmj.d5928 22008217 PMC3196245

[82] Gourlan M Bernard P Bortolon C Romain AJ Lareyre O Carayol M . Efficacy of theory-based interventions to promote physical activity. A meta-analysis of randomised controlled trials. Health Psychol Rev. (2016) 10:50–66. 10.1080/17437199.2014.981777 25402606

[83] Moher D Liberati A Tetzlaff J Altman DG PRISMA Group. Preferred reporting items for systematic reviews and meta-analyses: the PRISMA statement. PLoS Med. (2009) 6:e1000097. 10.1371/journal.pmed.1000097 19621072 PMC2707599

[84] Kipping RR Payne C Lawlor DA. Randomised controlled trial adapting US school obesity prevention to England. Arch Dis Childhood. (2008) 93:469–73. 10.1136/adc.2007.116970 18252756

[85] Bandura A. Social foundations of thought and action: A social cognitive theory. Englewood Cliffs, NJ, US: Prentice-Hall, Inc. (1986).

[86] Ryan RM Deci EL. Self-determination theory and the role of basic psychological needs in personality and the organization of behavior. In: Handbook of personality: Theory and research, 3rd ed. New York, NY, US: The Guilford Press; (2008) p. 654–78.

[87] Golden SD Earp JAL. Social ecological approaches to individuals and their contexts: twenty years of health education and behavior health promotion interventions. Health Educ Behav. (2012) 39:364–72. 10.1177/1090198111418634 22267868

[88] Krieger N. Theories for social epidemiology in the 21st century: an ecosocial perspective. Int J Epidemiol. (2001) 30:668–77. 10.1093/ije/30.4.668 11511581

[89] Richards EL Riner ME Sands LP. A Social ecological approach of community efforts to promote physical activity and weight management. J Community Health Nurs. (2008) 25:179–92. 10.1080/07370010802421145 18979329

[90] Epstein LH Roemmich JN Saad FG Handley EA. The value of sedentary alternatives influences child physical activity choice. Int J Behav Med. (2004) 11:236–42. 10.1207/s15327558ijbm1104_7 15657024

[91] Paing AC McMillan KA Kirk AF Collier A Hewitt A Chastin SFM. The associations of sedentary time and breaks in sedentary time with 24-hour glycaemic control in type 2 diabetes. Prev Med Rep. (2018) 12:94–100. 10.1016/j.pmedr.2018.09.002 30214853 PMC6134430

[92] Thivel D Tremblay A Genin PM Panahi S Rivière D Duclos M. Physical activity, inactivity, and sedentary behaviors: definitions and implications in occupational health. Front Public Health. (2018) 6:288. 10.3389/fpubh.2018.00288 30345266 PMC6182813

[93] Epstein LH Roemmich JN. Reducing sedentary behavior: role in modifying physical activity. Exerc Sport Sci Rev. (2001) 29:103–8. 10.1097/00003677-200107000-00003 11474956

